# Commentary: MagR Alone Is Insufficient to Confer Cellular Calcium Responses to Magnetic Stimulation

**DOI:** 10.3389/fncir.2018.00097

**Published:** 2018-11-12

**Authors:** Xiaoyang Long, Sheng-Jia Zhang

**Affiliations:** ^1^Department of Neurosurgery, Xinqiao Hospital, Army Medical University, Chongqing, China; ^2^Department of Biomedical Engineering, School of Medicine, Tsinghua University, Beijing, China; ^3^College of Life Sciences and Oceanography, Shenzhen University, Shenzhen, China

**Keywords:** magnetogenetics, ISCA1, optogenetics, GCaMP, neuromodulation, non-invasive brain stimulation

Pang et al. (2017) have raised reproducibility concerns about our ISCA-based magnetogenetic stimulation approach (Long et al., 2015). However, we strongly suspect that poor plasmid design and expression efficiency may explain their failure to reproduce our earlier findings, which demonstrated ISCA-based magnetic stimulation in seven systematic experiments carried out at the cellular, electrophysiological, circuit, and behavioral levels. In the following commentary, we highlight how flaws in the experimental design in their paper, and misinterpretations of our findings, introduce serious issues for the interpretation of their results.

The key to efficient magnetogenetic stimulation is the efficient delivery and expression of *Isca1*, previously renamed as MAR (Long et al., 2015). Pang and colleagues were not fully aware of how we designed our codon-optimized *Isca1* expression constructs, and thus their claim to have been using DNA constructs that are “almost identical” to ours is inaccurate. We clearly stated that we used a CAG promoter, which is much stronger than their EF-1α promoter. In the interests of promoting future investigation into the matter, we will openly share our codon-optimized cDNA constructs with the scientific community upon request. Although we are unaware of the source and DNA sequence of the EF-1α promoter used by Pang and colleagues, Zheng and Baum emphasized that different sources and sequences of EF-1α promoter jeopardize target specificity and gene expression (Zheng and Baum, 2014), which is in agreement with our initial pretesting on promoter-dependent effectiveness of *Isca1* for magnetogenetics. One likely explanation for such an apparent discrepancy is the large difference in expression level of *Isca1*.

Moreover, they performed many of their experiments after only 24 h of plasmid expression, rather than the 48 h post-transfection time window that we used. Their transfection efficiency (~30%) in HEK-293 cells was markedly lower than ours (~94%), and their transfection efficiency in neurons was poor (~3%) when compared to our population-wide infection (100%, Figures 1A,B) using our rAAV system (Zhang et al., 2007, 2013). In light of their limited *Isca1* expression under a weaker promoter and with a shorter expression time, we believe that their expression levels fell substantially short of ours, which is a very likely explanation for why they were unable to reproduce our earlier magnetogenetic stimulation results.

**Figure 1:**
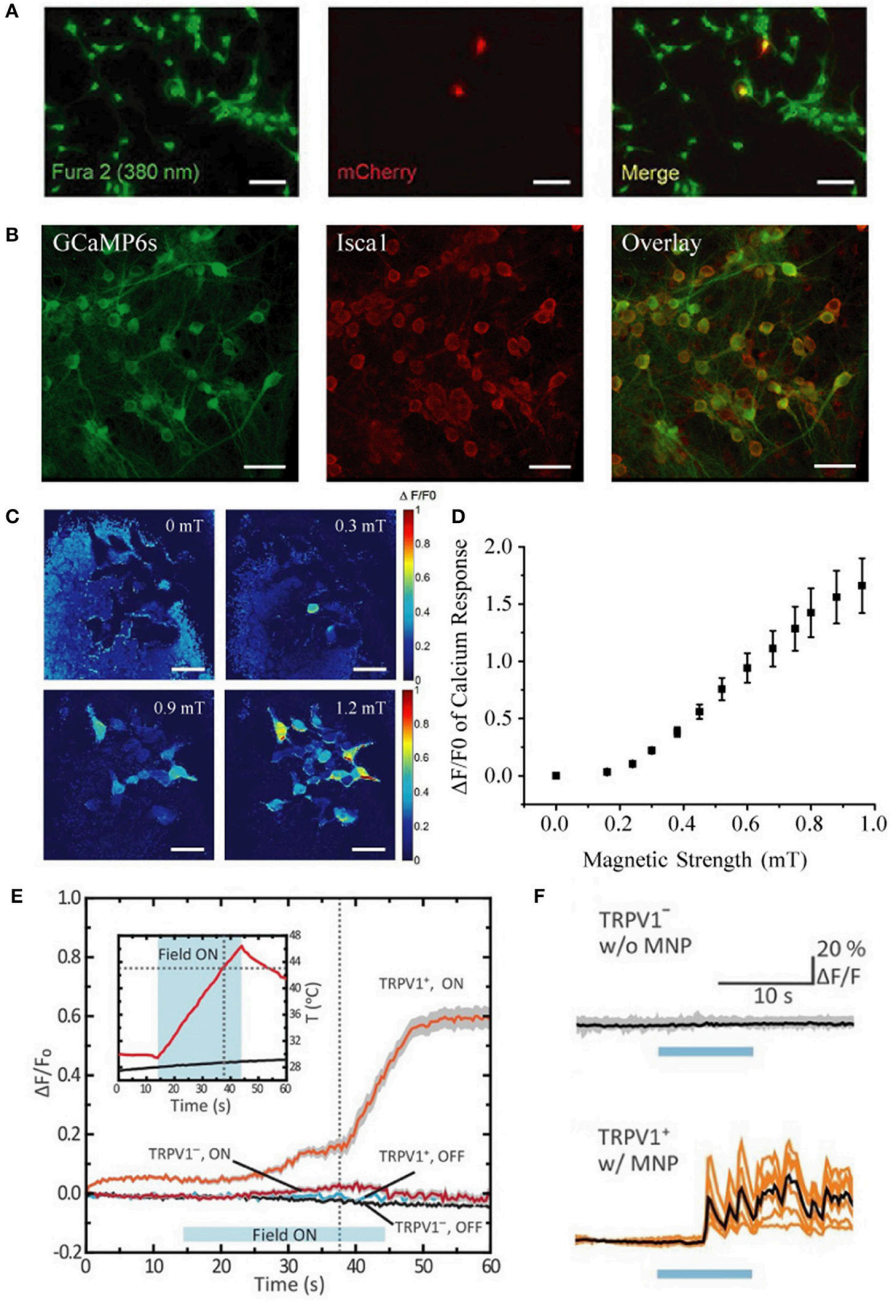
Comparison of transfection vs. infection efficiency in neurons, distinct calcium response patterns between HEK-293 cells and cultured neurons, and quantification of response threshold. **(A)** Extremely low transfection efficiency (~3%) of cultured hippocampal neurons with pLenti-EF1α-MagR-IRES-mCherry-3flag by Lu and colleagues [This panel was taken and modified from Figure 7A in *Frontiers in Neural Circuits* (Pang et al., 2017)]. **(B)** Nearly 100% coinfection of rAAV-CAG-*GCaMP6s* and rAAV-CAG-*Isca1* in cultured hippocampal neurons in our system. Scale bar, 50 μm. **(C)** Heat map showing normalized calcium fluorescence intensity change (ΔF/F0) of HEK-293 cells at 0, 0.3, 0.6, and 0.9 mT. Fluorescence increase was observed at 0.3 mT. Scale bar, 50 μm. **(D)** Averaged GCaMP6s fluorescence change in calcium signal (ΔF/F0) of 14 different groups of HEK-293 cells measured at various magnetic field strengths generated by a pair of electric coils. ΔF/F0 reached about 20% at 0.3 mT. **(E,F)** Different temporal kinetics of the magnetic control of cellular activity between HEK-293 cells and neurons [This panel was modified from Figure 1F and Figures 2G,H with permission from the *Science* magazine (Chen et al., 2015)]. Magnetothermal activation of nanoparticle-tethered heat-sensitive capsaicin receptor TRPV1 is stimulated in HEK-293 cells and cultured hippocampal neurons. The average onset latency is about 15 and 5 s for HEK-293 cells **(E)** and neurons **(F)**, respectively. Calcium response pattern in HEK-293 cells is similar to that observed with HEK-293 cells by us (Long et al., 2015), with a rising calcium response first and then a stabilizing calcium response to the externally applied magnetic field.

They inferred that our 350% increase in calcium influx was due to magnetic stimulation for “as long as 7 min.” In fact, our magnetic stimulus was applied for only 10–60 s, and it was the subsequent calcium activity that was measured for over 7 min. Furthermore, they expressed disbelief that we could evoke calcium responses with 1.0 mT. Actually, we varied the magnetic strength and found the threshold for detecting calcium response with GCaMP6s to be as low as 0.3 mT (Figures 1C,D), indicating that efficient expression of ISCA1 is the critical prerequisite in a functional magnetogenetic stimulation paradigm.

Furthermore, they raised concerns that our magnetic device “could raise the temperature of the cultured cells, leading to calcium changes.” However, this possibility could be ruled out for three reasons: First, all stimulation was performed at room temperature using static magnetic fields produced by electromagnetic coils or magnetic bars, which generate no power (and therefore introduce negligible heat); Second, no calcium increase was observed in ISCA1-negative cells by using the same setup; and Third, in TRPV-based magnetothermal strategies (Huang et al., 2010; Stanley et al., 2012) involving temperatures up to 43°C, the TRPV-negative cells exhibited no detectable calcium increases, suggesting that high temperature alone is insufficient to evoke calcium influx (Chen et al., 2015).

Pang and colleagues instead suggest that “the only difference was that we used an inverted microscope with ample air circulation, while the previous study used an upright microscope.” In fact, upright microscopes are very commonly used for calcium imaging *in vitro* and *in vivo*. Furthermore, action potentials were observed in our patch clamp experiments using an inverted microscope, suggesting that the type of microscope is irrelevant to magnetic activation.

They raised the possibility that “unhealthy cell state” may have contributed to “merely random firing.” This is incorrect for multiple reasons: (i) synchronous calcium responses were elicited; (ii) multiple rounds of rapidly reversible neuronal activation were time-locked to magnetic field presentation; (iii) spontaneous calcium transients in ISCA1-negative neurons were very infrequent when compared with evoked responses in ISCA1-positive cells; (iv) on-/off- and direction-dependent responses were detected by calcium imaging and patch-clamp recording; and (v) no significant differences were observed in intrinsic electrical properties between ISCA1-positive and ISCA1-negative neurons. This evidence strongly argues against their speculation of an “unhealthy cell state.”

They also suggested that “a sustained elevation of intracellular calcium could be an indication of an unhealthy state” referring to calcium responses in our HEK-293 cells that “continued to rise but never came down.” Surprisingly, they appeared unaware that similar patterns had been reported in HEK-293 cells in other prominent studies (Chen et al., 2015; Wheeler et al., 2016). Notably, transient calcium influx in neurons and saturating responses in HEK-293 cells may reflect differences in their cellular and electrophysiological properties or even their expression efficiency for ISCA1.

Furthermore, they emphasized without scientific rationale that the average calcium response onset latency of 7.8 s that we reported was “very long” and “very unusual” in that all neuronal stimuli “fall in the millisecond range.” In fact, onset latencies of existing magnetic actuators are all in the range of seconds rather than milliseconds. For instance, Chen et al. (2015) reported onset latencies of ~5 s and ~15 s for neurons and HEK-293-FT cells, respectively (Figures 1E,F). Considering the differences in physical properties between light and magnetic fields and in the biophysical features of light-sensitive vs. magnetism-sensitive proteins, it is not at all surprising that the temporal kinetics of magnetogenetics would be different from that of optogenetics.

While Pang and colleagues made laudable efforts to perform “positive control” applications of ATP or KCl to verify the calcium responses of their cells, unfortunately, the most important positive control is missing: comparisons, under their conditions of similarly low expression efficiency, of *Isca1* with the other four published magnetogenetic actuators, which include Ferritin-TRPV1, Ferritin-TRPV4, Nanoparticle-TRPV1, and EPG (Chen et al., 2015; Stanley et al., 2016; Munshi et al., 2017; Krishnan et al., 2018). Without this side by side comparison, the critical positive control for magnetic field generation that is sufficient to activate these levels of protein expression is lacking.

There were various other issues that may have impeded their ability to replicate our experiments properly. For example, they used the HEPES buffer instead of the commonly used calcium imaging buffer, which may have altered cellular excitability.

Since ISCA1-based magnetogenetics is still in its infancy, we hope that our discovery will stimulate further advances in this line of research (Knöpfel and Akemann, 2010). Given the arguments presented here, we believe that our key finding, that ISCA1 alone is sufficient to enable magnetic control of cellular activity, remains valid, and we are optimistic that future experimentation will confirm and build on the foundations of our work.

## Author contributions

All authors listed have made a substantial, direct and intellectual contribution to the work, and approved it for publication.

### Conflict of interest statement

The authors declare that the research was conducted in the absence of any commercial or financial relationships that could be construed as a potential conflict of interest.
